# Supplementary material for: Hand contamination and hand hygiene knowledge and practices among commercial transport users after the SARS-CoV-2 virus (COVID-19) scare, Enugu State, Nigeria
Source: PLOS Glob Public Health. 2024 May 31;4(5):e0002627. doi: 10.1371/journal.pgph.0002627 (PMC11142581; doi:10.1371/journal.pgph.0002627)
Supplement: S1 Table — (DOCX) [file pgph.0002627.s001.docx]

S1 Table: Varimax rotated principal component (PC) matrix of hand hygiene belief and practices among commercial transport users in Nsukka and Enugu towns, Enugu State, Nigeria.

| **Variables** | **Principal components (PC)*** | | | |
| --- | --- | --- | --- | --- |
|  | **1** | **2** | **3** | **4** |
| C6: After shaking hands with someone | 0.875 |  |  |  |
| C8: After removing my mask | 0.871 |  |  |  |
| C7: After touching animals or pets | 0.701 |  |  |  |
| C4: After I touch frequently used surfaces | 0.697 |  |  |  |
| D2: I do not believe that shaking hands with strangers can get me contaminated with harmful microorganisms | - | - | - | - |
| D3: I can get contaminated from harmful microorganisms if I touch my eyes, nose, or mouth with my unwashed hands, or not using hand sanitizer |  | 0.797 |  |  |
| D5: Do you believe practicing hand washing can help you fight COVID-19 |  | 0.795 |  |  |
| D1: I am protecting my health by washing my hands or using hand sanitizer |  | 0.762 |  |  |
| D4: My hand hygiene practice increased greatly after COVID-19 |  | 0.735 |  |  |
| C1: Before I eat or handle food |  |  | 0.758 |  |
| C2: After using a public toilet or urinal |  |  | 0.758 |  |
| C3: After blowing my nose, coughing, or sneezing |  |  | 0.712 |  |
| C5: When my hands are visibly dirty |  |  |  | 0.881 |
| **Eigenvalues** |  |  |  |  |
| Total | 3.326 | 2.387 | 1.491 | 1.099 |
| Variance (%) | 25.6 | 18.4 | 11.5 | 8.5 |
| Cumulative (%) | 25.6 | 43.9 | 55.4 | 63.9 |

PC1: Purposive/deliberate hand hygiene practices (sky blue); PC2: Hand hygiene belief (red); PC3: Habitual/customary hand hygiene practice (purple); PC4: When hands are visibly dirty. *Correlation values ≤ 0.39 were excluded.
