# Supplementary material for: Hand contamination and hand hygiene knowledge and practices among commercial transport users after the SARS-CoV-2 virus (COVID-19) scare, Enugu State, Nigeria
Source: PLOS Glob Public Health. 2024 May 31;4(5):e0002627. doi: 10.1371/journal.pgph.0002627 (PMC11142581; doi:10.1371/journal.pgph.0002627)
Supplement: S2 Table — (DOCX) [file pgph.0002627.s002.docx]

S2 Table: Classification matrix of purposive practice indicators (C4, C6, C7, C8) by the single generated binary purposive practice variable (Practice P50).

|  | Options | | | | |  |  |
| --- | --- | --- | --- | --- | --- | --- | --- |
|  | **Never** | **rare** | **Sometimes** | **Very often** | **Always** | **Total** |  |
| **C4** |  |  |  |  |  |  |  |
| Poor | 32 (5.3) | 131 (21.8) | 98 (16.3) | 31 (5.2) | 9 (1.5) | 301(50.2) | χ^2^ = 205.30 |
| Good | 4 (0.7) | 20 (3.3) | 95 (15.8) | 67 (11.2) | 113 (18.8) | 299 (49.8) | p < 0.0001 |
| **C6** |  |  |  |  |  |  |  |
| Poor | 168 (28.0) | 126 (21.0) | 7 (1.2) | 0 (0.0) | 0 (0.0) | 301(50.2) | χ^2^ = 380.59 |
| Good | 7 (1.2) | 62 (10.3) | 109 (18.2) | 44 (7.3) | 77 (12.8) | 299 (49.8) | p < 0.0001 |
| **C7** |  |  |  |  |  |  |  |
| Poor | 81 (13.5) | 106 (17.7) | 69 (11.5) | 31 (5.2) | 14 (2.3) | 301(50.2) | χ^2^ = 208.76 |
| Good | 17 (2.8) | 24 (4.0) | 54 (9.0) | 67 (11.2) | 137 (22.8) | 299 (49.8) | p < 0.0001 |
| **C8** |  |  |  |  |  |  |  |
| Poor | 195 (32.5) | 94 (15.7) | 10 (1.7) | 2 (0.3) | 0 (0.0) | 301(50.2) | χ^2^ = 316.17 |
| Good | 18 (3.0) | 81(13.5) | 100 (16.7) | 31 (5.2) | 69 (11.5) | 299 (49.8) | p < 0.0001 |
