# Supplementary material for: Hand contamination and hand hygiene knowledge and practices among commercial transport users after the SARS-CoV-2 virus (COVID-19) scare, Enugu State, Nigeria
Source: PLOS Glob Public Health. 2024 May 31;4(5):e0002627. doi: 10.1371/journal.pgph.0002627 (PMC11142581; doi:10.1371/journal.pgph.0002627)
Supplement: Attachment — Submitted filename: Review Plos Article._Manuscript PGPH-D-23-01961pdf.pdf [file pgph.0002627.s003.pdf]

REVIEW REPORT
Hand contamination and hand hygiene knowledge and practices among commercial transport users after the SARS-CoV-2 virus (COVID-19) scare, Enugu State, Nigeria
Manuscript PGPH-D-23-01961

1. Summary of the research and overall impression

This study combines a determination of the contamination of the hands of commercial transport users in the cities of Nsukka and Enugu and a survey of the knowledge and attitudes of these same people. This revealed the existence of contamination by both fungi, notably Aspergillus spp, and bacteria such as E coli and Enterobacter spp.

The methodology seems appropriate to us for the work carried out both for bacteriology and for the survey.

However, some weaknesses were noted, notably no reason as been estated to the limitation of the collection of hand swab and hand wash samples at 100. Also, no precise basis has been given to the direct consent of mature adolescent aged 14 to 17 without parental authorization. The presentation of the results is disparate and uncoordinated.

The discussion could have been more thorough and description of the media used to identify the pathogen could have been more summarize.

However, we can note that the methodology made it possible to meet the objectives set and to obtain results rarely found in our context. Another the limitation of this study is that this not emphasize pathogenic role of this isolated pathogens and the impact of the colonization in the hands in particular *Staphylococcus aureus* and *Salmonella sp*.

2. Discussion of specific areas for improvement

⇒ Major Issues

No major issues has been identified. However not being an expert for sampling, this has to been focused on

⇒ Minor issues

Some minor issues can be pointed out in this article.

1. The column remark can be removed from tables 2 & 3
2. All figures has been missing. Insert figures from1 to 9
3. Table 5: check the results for the education variable. Generally, respondents who have a primary level have the largest proportion. In this study this is quite the opposite. What explanation can be given to this fact?
4. Review the presentation of table 9
5. It is necessary to discuss the fungi revealed by the culture in one piece before discussing the bacteria identified. So, in microbial contamination section's : regroup all fungal contamination results then bacterial contamination results. No back and forth. In that sense:
    a. replace line 282-286 after line 275.
    b. Place table 4 as table 3 and vice versa
6. Line 279-280: replace sentence by "The fourth most prevalent bacteria species were *Escherichia coli* (16%), *Enterobacter spp.* (11%), *Proteus spp.* (8.0%), and *Stapylococcus aureus* (8.0%)"
7. Section Questionnaire responses (Demographics, Hand hygiene knowledge and Beliefs about hand hygiene)
    a. present the results regarding population of study first before the microbial contamination section's.
8. Line 369-376: editing issues
9. Line 370 define : "often", "rare", "sometimes"
10. In the discussion part
    a. emphasize the pathogenic role of hand swap sample with *Salmonella* isolated or *Staphylococcus*.
    b. Insist in the role of colonization by these pathogens bacteria and fungi in the discussion:
        i. the colonization by gram negative bacteria is more important in period of high temperature and humidity. That may explain these results since the study is conducted probably in the rainy season in these cities.

c. Explain the role of fungi in cross-transmission. Yeast colonization of hands are increasingly and a role in the cross-transmission.
d. Highlight the fact this study can be used to improve the availability of water and soap in Enugu and Nsukka to perform hand hygiene
