# Supplementary material for: Hand contamination and hand hygiene knowledge and practices among commercial transport users after the SARS-CoV-2 virus (COVID-19) scare, Enugu State, Nigeria
Source: PLOS Glob Public Health. 2024 May 31;4(5):e0002627. doi: 10.1371/journal.pgph.0002627 (PMC11142581; doi:10.1371/journal.pgph.0002627)
Supplement: Attachment — Submitted filename: Review Plos Article 2.pdf [file pgph.0002627.s005.pdf]

REVIEW PLOS ARTICLE

Hand contamination and hand hygiene knowledge and practices among commercial transport users after the SARS-CoV-2 virus (COVID-19) scare, Enugu State, Nigeria

Manuscript PGPH-D-23-01961

I had only minors issues on this article. Most of them has been corrected accordingly (see table below). From my part, this article is ready to be published

- **Minor issues corrections**

Minor Issues	Correction made by the authors
Removal of column remarks in tables 2 & 3	Done
Insertion of figures from1 to 9	Done
Population of study the characterization	Done
Presentation of fungi and bacterial	Done
Definition of variable: "Very", "often", "Sometimes", "Rarely"	Not characterized
Discussion on Pathogenic role of hand swap sample	Done
Role of fungi in cross-transmission	Explanation done
Consequences of the colonization by bacteria and fungi	Done
