# Supplementary material for: Hand contamination and hand hygiene knowledge and practices among commercial transport users after the SARS-CoV-2 virus (COVID-19) scare, Enugu State, Nigeria
Source: PLOS Glob Public Health. 2024 May 31;4(5):e0002627. doi: 10.1371/journal.pgph.0002627 (PMC11142581; doi:10.1371/journal.pgph.0002627)
Supplement: Attachment — Submitted filename: Response to Reviewers.docx [file pgph.0002627.s004.docx]

Response to Reviewers:

Hand contamination and hand hygiene knowledge and practices among commercial transport users after the SARS-CoV-2 virus (COVID-19) scare, Enugu State, Nigeria

ID: Manuscript PGPH-D-23-01961

**Comments to the Author**

1. Does this manuscript meet PLOS Global Public Health’s [publication criteria](https://journals.plos.org/globalpublichealth/s/criteria-for-publication)? Is the manuscript technically sound, and do the data support the conclusions? The manuscript must describe methodologically and ethically rigorous research with conclusions that are appropriately drawn based on the data presented.

Reviewer #1: Yes

Reviewer #2: Yes

2. Has the statistical analysis been performed appropriately and rigorously?

Reviewer #1: I don't know

Reviewer #2: No

**Authors’ Answer**: The minor concern raised about non-inclusion of comparison of proportion in abstract has been addressed in appropriate section below. Additional comment raised concerning 3D figures have been addressed at appropriate locations below. Apart from these two minor issues, there were no other issues raised suggestive of a faulty statistical analysis. Additionally, even though neither of the reviewers suggested a need for additional analysis, we explored additional statistical analysis. We had to generate single variable measures for “purposive hand hygiene practice”, “customary hand hygiene practice”, and “positive hand hygiene belief” so we could compute odd ratios for good hand hygiene practices and belief in relation to demographics using binary logistic regression. We have included this in the data analysis and results section (including the accompanying supplementary materials). We believe the newly added results has provided additional summary to the knowledge, attitude and practice section. However, we believe that with or without the additional results, the objectives of the study have been sufficiently addressed.

3. Have the authors made all data underlying the findings in their manuscript fully available (please refer to the Data Availability Statement at the start of the manuscript PDF file)?

The [PLOS Data policy](https://journals.plos.org/globalpublichealth/s/data-availability) requires authors to make all data underlying the findings described in their manuscript fully available without restriction, with rare exception. The data should be provided as part of the manuscript or its supporting information, or deposited to a public repository. For example, in addition to summary statistics, the data points behind means, medians and variance measures should be available. If there are restrictions on publicly sharing data—e.g. participant privacy or use of data from a third party—those must be specified.

Reviewer #1: Yes

Reviewer #2: Yes

4. Is the manuscript presented in an intelligible fashion and written in standard English?

PLOS Global Public Health does not copyedit accepted manuscripts, so the language in submitted articles must be clear, correct, and unambiguous. Any typographical or grammatical errors should be corrected at revision, so please note any specific errors here.

Reviewer #1: Yes

Reviewer #2: Yes

5. Review Comments to the Author

Please use the space provided to explain your answers to the questions above. You may also include additional comments for the author, including concerns about dual publication, research ethics, or publication ethics. (Please upload your review as an attachment if it exceeds 20,000 characters)

**Reviewer 1**

**1. Summary of the research and overall impression**

This study combines a determination of the contamination of the hands of commercial transport users in the cities of Nsukka and Enugu and a survey of the knowledge and attitudes of these same people. This revealed the existence of contamination by both fungi, notably Aspergillus spp, and bacteria such as E coli and Enterobacter spp. The methodology seems appropriate to us for the work carried out both for bacteriology and for the survey. However, some weaknesses were noted, notably no reason as been estated to the limitation of the collection of hand swab and hand wash samples at 100. Also, no precise basis has been given to the direct consent of mature adolescent aged 14 to 17 without parental authorization. The presentation of the results is disparate and uncoordinated. The discussion could have been more thorough and description of the media used to identify the pathogen could have been more summarize. However, we can note that the methodology made it possible to meet the objectives set and to obtain results rarely found in our context. Another the limitation of this study is that this not emphasize pathogenic role of this isolated pathogens and the impact of the colonization in the hands in particular *Staphylococcus aureus* and *Salmonella sp*.

**Authors’ response**: (1) **100 hand swap sample**: We believe the sample size of 100 hand swap sample is suitable for epidemiological survey on the presence or not of fungal and bacteria contamination of hands of commercial transport users. Casals-Pascual et al. (2020; <https://doi.org/10.1053/j.gastro.2019.11.305>) showed that a sample size of 55 (~ 110 samples for two groups comparison) has adequate statistical power (80%) at 5% level of significance for microbial diversity studies. According to their estimates, even a sample size of 20 is good enough, but it is relatively underpowered. See also, Ferdous et al. (2022; <https://doi.org/10.1038/s41385-022-00548-1>). (2) **Direct consent of mature adolescent, 15 – 17 year old without parental authorization**: The study procedure posed no or minimal risk to participants, thus matured adolescents of 15 to 17 who consented were allowed to participate without obtaining additional parental/guardian assent as approved by the ethical committee. The Child Right Act in Nigeria provides that mature adolescent has the right to give consent for scientific investigation without parental consent; even in national HIV prevalence survey 15 years old adolescent are regularly engage (Federal Ministry of Women Affair, Nigeria, 2004; Folayan et al., 2015; <https://doi.org/10.1111/dewb.12061>). (3) **Presentation of result disparate**: We understand that the review prefer that we present the result on fungi contamination (i.e. single and co-contamination) first before results on bacteria, and that results on demographic should come before those of contamination. We have now re-arrange as the reviewer suggested. Whatever will make the readers more comfortable is welcome. (4) **Discussion being more thorough**: We thank the review for making suggestions for improvement of some aspects of the discussion. Rightly so, all concerns raised concerning the discussion has been addressed; however, there is one of the suggestions concerning climatic condition and Gram negative bacteria colonization where we preferred to be more conservative. The reason for that is explained below. Additionally, we also looked at the concerns raised about references to description of media in the discussion; we have now addressed that by removing all such where they are not so relevant. (5) **Emphasis on pathogenic roles**: The emphasis have now been provided including the roles of fungi in cross-transmission. Including for *Staphylococcus aureus*, *Salmonella* spp., *Clostridium difficile*, *Candida albican*, etc.

**2. Discussion of specific areas for improvement**

Þ **Major Issues**

No major issues has been identified. However not being an expert for sampling, this has to been focused on.

**Author’s response**: We believe the sample size of 100 for hand swap sample is suitable for epidemiological survey on the presence or not of fungal and bacteria contamination of hands of commercial transport users. Casals-Pascual et al. (2020; <https://doi.org/10.1053/j.gastro.2019.11.305>) showed that a sample size of 55 (~ 110 samples for two groups comparison) has adequate statistical power (80%) at 5% level of significance for microbial diversity studies. Even sample size of 20 is good enough, but it is relatively underpowered. Also, Ferdous et al. (2022; <https://doi.org/10.1038/s41385-022-00548-1>)

Þ **Minor issues**

Some minor issues can be pointed out in this article.

1. The column remark can be removed from tables 2 & 3

**Authors’ response**: The “Remark” column in Table 2 and 3 has now been replaced with “Pathogenicity” after deliberation whether or not we should add it as a supplementary material. The “Pathogenicity” heading has been so used in other studies (e.g. Park et al. 2022, “Bacterial and fungal isolation from face masks under the COVID-19 pandemic. *Scientific Reports* 12: 11361. <https://doi.org/10.1038/s41598-022-15409-x>”). Also, references to the pathogenicity rating have been included.

2. All figures has been missing. Insert figures from1 to 9

**Authors’ response**: All the figures were attached at the end of the manuscript as per the journal instruction to authors. We apologise to the reviewer if they were misled into believing there were no figures while the figures were actually attached after the references.

3. Table 5: check the results for the education variable. Generally, respondents who have a primary level have the largest proportion. In this study this is quite the opposite. What explanation can be given to this fact?

**Authors’ response**: Indeed the reviewer made a very important observation. However, Nsukka is a university community and some of the parks are much patronised by students. Some parks in Enugu town were also patronised significantly by university students during the study period. Also, literacy rate in Enugu State is high. It is not uncommon for us to find higher frequency of secondary and tertiary education in studies were samples were collected in urban areas. The pattern changes when samples are collected mostly from the rural areas. But even in some of the rural areas, we tend to find more people who had at least secondary school education. From my previous studies in Enugu State, I do not find the distribution of the education unusual.

4. Review the presentation of table 9

**Authors’ response**: A little adjustment has been made to the Table 9. The sentence, “When I fail to wash my hands, what is the most common reason?” has now been corrected to read, “The most common reasons for failing to wash ones hands”.

5. It is necessary to discuss the fungi revealed by the culture in one piece before discussing the bacteria identified. So, in microbial contamination section’s: regroup all fungal contamination results then bacterial contamination results. No back and forth.

In that sense:

a. replace line 282-286 after line 275.

b. Place table 4 as table 3 and vice versa

**Authors’ response**: We are glad the reviewer pointed this out. We did not notice that the arrangement may not have been suitable for readers as we had intended to discuss single infection before co-infections. But we have now re-arranged as the reviewer suggested. Table 4 is now the new Table 3, and the previous Table 3 has now become Table 4. Any re-arrangement that would make the readers more comfortable is always welcome.

6. Line 279-280: replace sentence by “The fourth most prevalent bacteria species were *Escherichia coli* (16%), *Enterobacter spp.* (11%), *Proteus spp.* (8.0%), and *Stapylococcus* *aureus* (8.0%)”

**Authors’ response**: Thank you so much for this observation. *Enterobacter* spp. (10%) was erroneously ignored in the sentence while it was there in the Table. The error has been corrected.

7. Section Questionnaire responses (Demographics, Hand hygiene knowledge and Beliefs about hand hygiene)

a. present the results regarding population of study first before the microbial

contamination section’s.

**Authors’ response**: The results about demographics has now been presented first. Table 5 has now become Table 2, and previous Tables 2, 3, 4 re-arranged accordingly. We also believe it is better that way as we have now moved the sample size from the methods section to the start of the results section.

8. Line 369-376: editing issues

**Authors’ response**: The section previously line 369 – 376 has been edited for clarity.

9. Line 370 define : “often”, “rare”, “sometimes”

**Authors’ response**: “always”, “very often”, “sometimes”, “rare”, “never” are standard English terminologies that are used in everyday conversations. We did not see any reason to define a new meaning for the terms, nor did we see any need to redefine their meaning to the study participants. They were not challenging to understand during the pretesting of the questionnaires. Also, these ordinal comparatives are standard terms used in questionnaires, especially Likert type questions. However, below are the definitions/meaning implied:

Always – at all times

Very often – regularly, again and again, often, customarily, habitually.

Sometimes – occasionally

Rare – infrequent, few and far between, unusual, uncommon.

Never – at no time, not ever, not at all.

10. In the discussion part

a. emphasize the pathogenic role of hand swap sample with *Salmonella* isolated

or *Staphylococcus*.

**Authors’ response**: *Staphylococcus*, *Salmonella* and *Clostridium* pathogenic roles have now been more emphasised as suggested by the reviewer.

b. Insist in the role of colonization by these pathogens bacteria and fungi in the

discussion:

i. the colonization by gram negative bacteria is more important in period of high temperature and humidity. That may explain these results since the study is conducted probably in the rainy season in these cities.

**Authors’ response**: The commercial transport users hands were colonised by both Gram positive and Gram negative bacteria. Indeed this study was done during the rainy season, and it is probable that the colonization of the Gram negative bacteria is related to high temperature and humidity that characterise the rainy season in Nigeria. We could also assume that the ability of *Clostridium* resistant spores to withstand harsh conditions could have contributed to contamination of hands of the commercial transport users. However, we prefer to be cautious with making assumptions. We prefer to be more conservative with assumptions that we do not have data to support from our study. We did not record the climatic conditions within the study periods, and there was no intention to ascertain the effect of seasonality on contamination level; while that may be interesting, we believe research is a continuum. We plead with the reviewer to allow us to be a little more cautious here.

c. Explain the role of fungi in cross-transmission. Yeast colonization of hands are

increasingly and a role in the cross-transmission.

**Authors’ response**: We have now included discussion on cross-transmission and other additional risks posed by yeasts. We believe that the addition further the risks of the pathogens, and emphasizes the public health significance of the study.

d. Highlight the fact this study can be used to improve the availability of water and soap in Enugu and Nsukka to perform hand hygiene

**Authors’ response**: This has now been highlighted in the discussion. However, why we acknowledge that provision of water and soap or hand sanitizers may potentially improve hand hygiene practice, we believe it is not a sufficient intervention in itself.

Reviewer #1: this article meets the publication criteria of the journal. The english is done in a very good writing and is very clear. The methodology used on my knowledge alllows the results to be probant. However, for the sampling aspect of this study, an epidemiologist has to answer if the number of hand swab chosen is the one to do.

**Authors’ Response**: We believe the sample size of 100 for hand swap sample is suitable for epidemiological survey on the presence or not of fungal and bacteria contamination of hands of commercial transport users. Casals-Pascual et al. (2020; <https://doi.org/10.1053/j.gastro.2019.11.305>) showed that a sample size of 55 (~ 110 samples for two groups comparison) has adequate statistical power (80%) at 5% level of significance for microbial diversity studies. Even sample size of 20 is good enough, but it is relatively underpowered. Also, Ferdous et al. (2022; <https://doi.org/10.1038/s41385-022-00548-1>)

**Reviewer #2**

Reviewer #2: We consider this to be an interesting study that respected ethical rules. The recommendations from this study can have a positive impact at the community level. However, please find below some comments:
- Please, briefly describe the objectives in the abstract

**Authors’ Answer**: The aim and objective are captured in this now corrected sentence, “Hence, assessment of commercial transport users’ knowledge and practices on hand hygiene, and potential contamination with infectious agents which is the aim of the study, aligns with general health concern of quantifying contamination risk levels to predict disease outbreaks”

- Please, briefly describe the statistical method for data analysis

**Authors’ response**: The abstract is already over 300 words. Adding a description of the statistical analysis could take it to over 400. We understand the reviewer’s desire to have the statistical analysis stated in the abstract, but we prefer not to add it to the abstract. However, if the reviewer meant addition of description into the “statistical analysis” in the material and methods, then we have done as the reviewer requested.

- The results of the statistical comparison of proportions must be included in the abstract

**Authors’ response**: Statistical comparison of proportion has been included in the abstract as suggested by the reviewer.

- Comments should not be included in the results section (both in the summary and the full manuscript). Please just provide results

**Authors’ Answer**: Comments in the results have been removed (e.g. “It was difficult to get pure isolates of some species”). Additionally, the “Remark” column in Table 2 and 3 has now been replaced with “Pathogenicity” after deliberation whether or not we should add it as supplementary material. It has been so used in other studies (e.g. Park et al. 2022, “Bacterial and fungal isolation from face masks under the COVID-19 pandemic. *Scientific Reports* 12: 11361. <https://doi.org/10.1038/s41598-022-15409-x>”). Also, references to the pathogenicity rating have been included. It is our desire to specify the pathogenicity of the isolates.

- In the introduction :
We think you can reduce the length of the text
The objectives must also be well worded (by including not only hand hygiene practices and hand contamination level among commercial park users, but also the comparison of contamination between the two cities)

**Authors’ response**: The length of the introduction has been reduced further. Sentences considered redundant have been removed. Also, the objective has now been more clearly worded. It now reads, “This study sought to provide concrete information on hand contamination level and hand hygiene knowledge, attitude and practices among commercial park users in Nsukka and Enugu, and to compare the contamination level between the two cities.”

- In the methodolgy section, please explain the method of randomization of the five parks and the participants from each park included in the study

**Authors’ response**: A simple random lottery method sampling was used to select the parks.

- In the methodology section, line 55 : you should calculate the sample size using the SCHWARTZ formula and by maximizing the proportions p and p - 1 to 50% (the sample size will then be 384) and add the 10% of non-respondents. Wait until the results are written to enter the size sample of your study (600).

**Authors’ response**: The procedure for sample size computation was exactly what we used. We have now made clarification to the section, and included the equation for clarity. Also, the “600” sampled have now been moved to the results section.

- int the methodology, line 155 : "non inclusion critaria" is more relevant than "exclusion critaria"

**Authors’ response**: Done. “Exclusion” replaced with “non-inclusion”.

- in the results section : Confusion biais are possible in the comparison of microbial contamination between the two cities because we have no idea about how the participants included were randomized.

**Authors’ response**: As the reviewer rightly highlighted, a confusion bias can occur when a sampling design systematically distorts results. We designed the study to avoid any such systematic bias. Apart from the districts (Nsukka and Enugu) which were intentionally/purposively chosen, all other sampling procedure was done using standard statistically acceptable procedures. First the parks were chosen by simple random lottery method. The names of all the major/active commercial parks were listed, and the five picked while blindfolded were the parks sampled for each of the districts. The time of sample was also chosen to eliminate any chance of bias. Sampling was done within specified periods time periods daily. Alternation between sampling Nsukka and Enugu were also randomly selected. As it would have been impossible for us to collecting sample in Enugu and Nsukka at the same time, random picks of districts to sample for a given week was used to prevent bias due to sampling time. Also, hands to sample was the dominant one so as to prevent confusion bias. The study participants were also selected to prevent confusion bias. Randomization of participants was done by selecting every third commercial transport user that walks past a member of the research team that was positioned at the main entry into the parks. A similar procedure has been used by other authors (e.g. De Alwis etal., 2012. “A study on hand contamination and hand washing practice among medical students. *International Scholarly Research Notices*, <https://doi.org/10.5402/2012/251483>”). However, at some parks, it was difficult to identify which is the main entry point as there were multiple entry channels. In such places, the sentinel was positioned at a busy point within the park where park users were very likely to pass through before boarding a vehicle. Those who accepted to participate and met the inclusion criteria were thus included. The first ten swap samples collected using the sampling procedure was used. For the questionnaire, it was intended that 60 samples would be collected from each, but from pilot study, it was obvious that this was not realistic as traffics differ at the parks. Some parks were very busy, while some were not. Therefore, in parks were it was unrealistic to complete 60 questionnaires within the study periods, the leftover questionnaires were completed in one randomly selected of the four remaining parks. Where the selected park was not feasible, another was selected from the remaining three parks, and so on. But overall, the questionnaire sampling was well spread across the 5 parks per district as only one park was unable to meet the 60 person target, but met 80%.

In addressing the reviewer’s concern, we have now stated how the participants were randomized more clearly. It was erroneously omitted during the preparation of the manuscript for publication.

-It should be interesting to compare microbial contamination regarding demographic variables (sexe, level of education, marital statut, etc.) and Hand hygiene knowledge.

**Authors’ response**: Yes. It would have been interesting to compare microbial contamination at demographic level. Additionally, though not mentioned by the reviewers, it would also have been interesting to assess the risks (odd ratio or risk ratios) of contamination in relation to hand hygiene knowledge, attitude and practices. But the authors intentionally decided not to explore those options. For every researcher, any additional result is always helpful: it could help improve policy formulation or even help save some lives. But in research design there is always a trade-off on some information in order to keep cost of research within a feasibility threshold especially for self-sponsored research such as this one. The hand contamination was done for 100 people, and we fear it may not be able to provide comprehensive and robust information on demographic level risks from statistical analysis. However, the additional information not provided did not in any way compromise on the sanctity of the three core objectives of the study which were fully addressed.

- avoid 3D figures

**Authors’ response**: We believe the heatmaps provide easy summary of the microbes by parks prevalence. We believe Tables and Figures provide a balance to result presentation. Tables allow for detailed check of the numbers while figures allow a more relaxed cursory look through the graphs. Also, it would be difficult to present two 12 columns by 21 rows tables that would be as appealing to read through as heatmaps.
